# Engaging businesses and faith-based organizations in public health interventions: Lessons learned from a COVID-19 and flu vaccine detailing program in the Northeast Bronx^☆^

**DOI:** 10.1016/j.puhip.2022.100353

**Published:** 2022-12-13

**Authors:** Fatoumata Diallo, Lissette Paulino, Lauren J. Shiman, Kim Freeman, Brandon Brooks, Diana Banson, Anita Reyes

**Keywords:** Health promotion, Vaccine hesitancy, Community engagement, Program evaluation

## Abstract

**Objectives:**

The NYC Department of Health and Mental Hygiene conducted a COVID-19 and flu vaccine community detailing program to influential businesses and faith-based organizations in the Northeast Bronx in 2022 to increase COVID-19 and flu vaccine knowledge and uptake among residents of the area.

**Study design:**

program evaluation.

**Methods:**

The program was piloted in the Northeast Bronx, a geography selected based on prior low COVID-19 and flu vaccination rates and high COVID-19 case positivity rates. Barbershops, hair salons, beauty salons, nail salons and faith-based organizations were selected as potential partners because their owners or staff typically spend at least an hour in interactions with clients. From January 2022 through April 2022, two detailing visits were conducted by engagement staff: an initial visit to all potential partners in the selected geography, and a follow up visit to those who committed to be champions of health.

**Results:**

Out of 113 identified businesses/organizations, 70 met the criteria to be potential partners in the program. After being contacted by health department staff, 45 (64%) potential partners committed to be champions of health. During the four months of the pilot, zip codes with the highest level of program engagement experienced greater percent increases in COVID-19 vaccination rates during the program period compared to NYC and Bronx averages. Flu vaccination rates during the program period were not available.

**Conclusion:**

Supplementing other local public health efforts, the community detailing pilot program demonstrates a model of dissemination of health information through local business leaders, and provides lessons learned to increase champion commitment.

## 1 Introduction

New York City's Department of Health and Mental Hygiene (NYC Health Department) has used place-based health initiatives to support neighborhoods unjustly burdened with premature morbidity and mortality. After many years of developing place-based initiatives and building strong relationships in three historically excluded neighborhoods with high premature mortality, the NYC Health Department launched Bureaus of Neighborhood Health in these communities [1]. Situated in the South Bronx, the Bureau of Bronx Neighborhood Health aims to promote health equity and reduce health disparities through place-based initiatives, research, and programming.

Generations of structural racism, including the lasting impacts of redlining, have resulted in the Bronx having a high burden of health challenges and premature deaths. This vibrant and diverse borough is home to over 1.4 million residents; 56.0% identify as Hispanic or Latino, 38.6% identify as Black, and 34.6% were born outside of the United States [2]. It has also been ranked the least healthy county in the state annually since the *County Health Rankings* launched in 2010 [3]. Pre-existing chronic conditions, overcrowded housing, and other health and socio-economic factors have made Bronx residents more vulnerable to the direct and indirect impacts of the COVID-19 pandemic [4]. COVID-19 cases, hospitalizations, and death rates in the Bronx are among the highest in NYC [5]. Hispanic and Black residents, who are highly represented in the Bronx, have been disproportionately affected by the COVID-19 pandemic [6]. Nevertheless, between 75% and 82% of residents living in the Northeast Bronx received at least one dose of a COVID-19 vaccine, compared to 88% of residents across NYC [7]. Reduced vaccination rates might stem from historical examples of oppression which has led to residents’ public distrust of government and scientific sources. Hence, providing accurate information about the COVID-19 vaccine through direct engagement of at-risk Bronx communities is critical.

The NYC Health Department has operated a Public Health Detailing program since 2003 to engage healthcare providers in sharing information about specific health topics with their patients. Modeled after pharmaceutical detailing, the Public Health Detailing program was designed to address gaps in provider knowledge and promote the use of essential preventive services by primary care providers [8]. Detailing efforts by the NYC Health Department to primary care providers have shown to increase knowledge and implementation of recommended practices [8,9], as well as improve clinical management, lifestyle modification, and behavior change [10].

To reach historically excluded populations burdened with preventable health disparities, trusted health information may come from community leaders beyond healthcare providers. Previous health promotion interventions have engaged barbershops, hair salons, and places of worship; these community-based businesses and organizations are highly accessible and are considered to be safe spaces by community members. A systematic review of obesity interventions in African American faith-based organizations (FBOs) found improvements in participants’ weight and health behaviors [11]. Barbershops and salons have been shown to increase recommended health screenings and improve chronic disease management in community members [12,13]. A literature review of health promotion conducted in salons and barbershops found that businesses reported already sharing important health information with their customers, and that their customers were interested in receiving the health information from their stylists or barbers or in salons or barbershops [14].

To address vaccine hesitancy and increase vaccination rates, the Bureau of Bronx Neighborhood Health piloted a community detailing program in the Northeast Bronx. The program addressed both COVID-19 and flu vaccines since the detailing took place during flu season and there are symptom similarities between the two viruses. The program met with business leaders while NYC's Public Health Detailing program detailed healthcare providers in the same neighborhoods to saturate the Northeast Bronx community with accurate information about COVID-19 and flu vaccines. To the authors' knowledge, this is the first public health detailing program to jointly engage health providers *and* business leaders in public health promotion. This article describes the community detailing model and program evaluation results, and explores lessons learned.

## 2 Methods

### 2.1 Program planning

#### 2.1.1 Developing list of potential champions

**Fig. 1:**
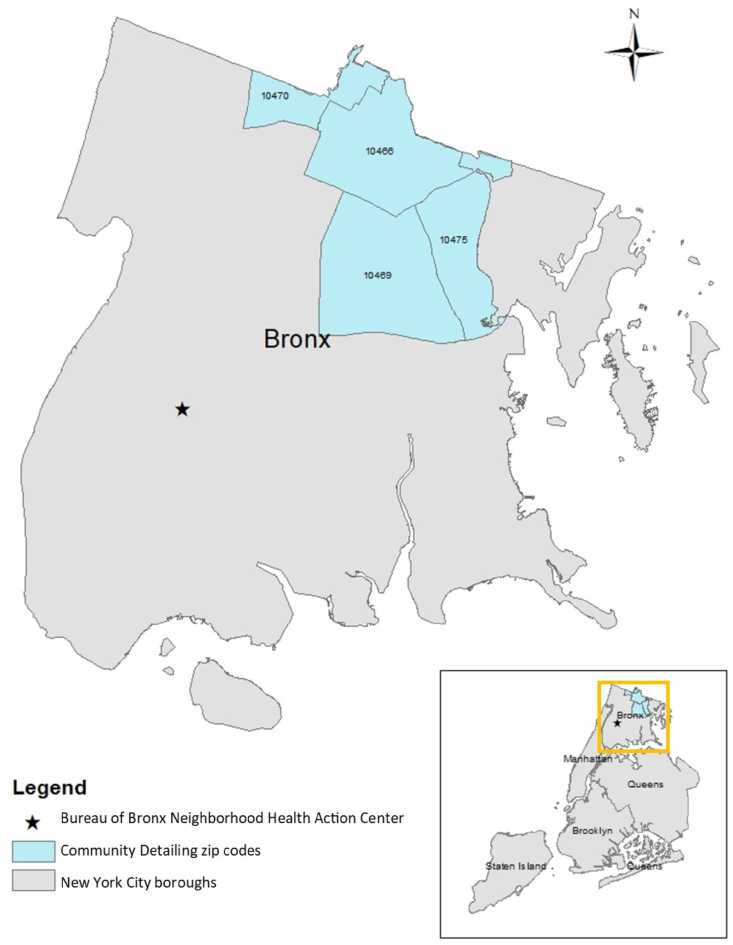
Map of the COVID-19 and flu vaccines community detailing program area.

As shown in Fig. 1, four zip codes in the Northeast Bronx (10466, 10469, 10470, 10475) were selected for the intervention based on low flu vaccination rate data from the 2020 NYC Community Health Survey and low COVID-19 vaccination [15] and high COVID-19 case positivity rates as of September 2021 [16]. The program selected barbershops, hair salons^1^, beauty salons^2^, nail salons, and FBOs as potential champions because these businesses are highly accessible by their communities and consistently spend at least an hour interacting with clients or community members. Businesses were identified through merged and deduplicated lists from Google Maps, the NY State Barber License, and the NYC Health Department's salesforce platform, Public Health Partners Connect. During initial visits, businesses or places of worship marked as temporarily or permanently closed, and those found to be in residential buildings, were excluded from the list. A map was created in GIS Mapping Software (ESRI). Initial and follow-up surveys were programmed into a data collection application (Survey123) and integrated into the ESRI map.

#### 2.1.2 Preparing detailing staff and materials

The NYC Health Department's Public Health Detailing program team conducted two days of intensive training for the community detailing project team, a bilingual Bronx-based team of community health workers and health promoters with members fluent in both English and Spanish. The training guided staff on how to introduce and frame the issue to businesses, how to provide recommendations and promote materials, how to overcome barriers and objections during visits, and how to gain commitments and close the visit.

Project materials disseminated during initial visits included: a letter from the NYC Commissioner of Health, informational flyers, brochures, and palm cards on COVID-19 and flu. At the initial visit, potential partners were also provided with personal protective equipment (PPE including surgical masks, KN95 and N95 masks, and hand sanitizer) and at-home COVID-19 test kits.

### 2.2 Program implementation

Project team members were assigned to specific neighborhood areas to minimize travel between visits. The detailing visits began at the end of December 2021. However, because of a COVID-19 surge and businesses’ limited capacity to engage during the December holiday season, the project was paused and relaunched in mid-January 2022. Visits occurred between January–April 2022.

During the initial visit, the team requested to speak with a decision-maker and provided an introduction and program overview. The project team member administered an oral survey and recorded responses into Survey123, collecting information about community perceptions of the COVID-19 and flu vaccines and documenting the partner's commitments to champion the importance of these immunizations. Businesses were invited to become champions of health by committing to one or more of the following: (1) speaking with their customers about the COVID-19 and flu vaccines and offering supporting materials to help answer any questions of concerns, (2) displaying posters and brochures that encourage vaccination, (3) hosting a Health Department staff person to table at the business to share information and answer customer's questions, or (4) any other activity suggested by the business relevant to COVID-19 and flu vaccine promotion.

Between visits, the team completed requests including providing additional resources or PPE and tabling at the champion's site to communicate directly with clients and passing residents. A month after the initial visit, sites that agreed to become champions of health received a follow-up visit and were invited to complete a follow-up survey to learn about their experiences participating in the program.

## 3 Results

### 3.1 Engagement in the program

#### 3.1.1 Identified partners

**Fig. 2:**
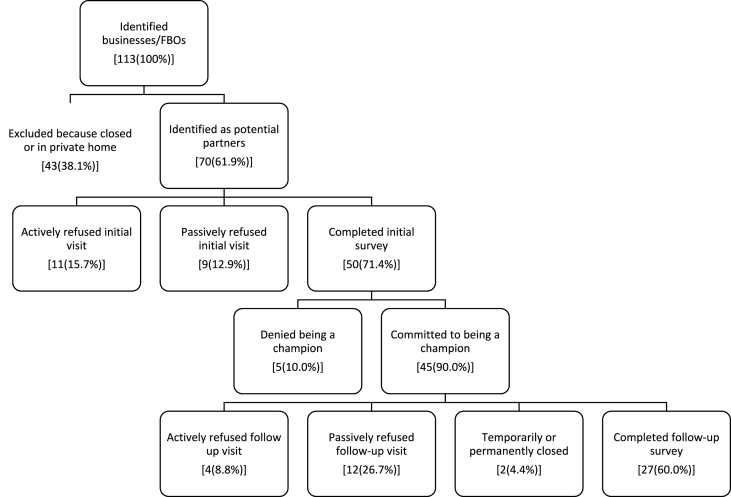
Business/FBO engaged in the COVID-19 and flu vaccines community detailing program [N(%)].

Fig. 2 details business/FBO engagement throughout the community detailing program. A total of 113 unique barbershops, hair salons, beauty salons, nail salons, and FBOs were identified. Of these, 43 were excluded during initial visits because they were found to be in a private home, temporarily or permanently closed, or had hours of operation that were incompatible with the project, yielding 70 potential partners. Most excluded sites were FBOs (n = 29) which had closed their sites and were only conducting online worship.

During the initial visit, twenty sites refused participation in the program (15.7%). Among those who committed as champions, sixteen sites refused the follow-up visit (8.8% actively, 26.7% passively) and two were found to be closed after multiple visit attempts. When sites refused to participate or complete the program, the community detailing teams were able to engage with them about the reason for their refusal. Sites that actively refused visits expressed distrust in government or stated that there were too many regulations at the time and were not sure if the detailing staff intentions were motivated by health or regulatory concerns. Sites were considered to have passively refused participation if they said that they were busy and did not have time to speak with the detailing staff, and that there was no good time to visit, or they were not sure when they owner would come in. When the detailing teams introduced the possibility of speaking about other health topics distinct from COVID-19 or flu vaccines in the future, most sites who refused participation in this project were willing to have health department staff attempt future engagement.

#### 3.1.2 Commitments to be champions of health

In total, 45 of the 70 potential partners committed to being champions of health. However, only 27 (60.0%) fully completed the program by agreeing to a follow-up visit and participating in the follow-up survey. Although one FBO agreed to be a champion and followed through on its commitment to host a health department staff, it passively refused the follow-up survey and consequently did not complete the program. As shown in Table 1, most sites that committed to being champions elected to display materials in their site (74.0%), though many committed to have vaccine conversations and offer materials (44.0%), or to host a health department staff person for tabling (34.0%). None suggested an alternate activity, an option included to create a more equitable partnership and give sites greater agency as program participants. Almost all sites accepted materials to distribute to residents (95%); 84.0% accepted flu posters and flyers and 74.0% accepted COVID-19 posters and flyers.

**Table 1:** Sites commitments to be champions of health and their adherence to the commitments.

	Sites That Made Commitment N(%)^a^	Sites Documented as Completing Commitment N(%)^b^
**Finishing program:** engaging in follow up visit and completing survey	45(90.0)	27(60.0)
**Commitment 1:** Having vaccine conversations and offering supporting materials	22(44.0)	8(36.4)
**Commitment 2:** Displaying posters and brochures that encourage vaccination	37(74.0)	7(18.9)
**Commitment 3:** Hosting a Health Department staff person to table at the business	17(34.0)	13(76.5)

a Represents results from the 50 surveyed businesses/FBOs during the initial visit.

b Completing commitments 1 and 2 could only be assessed among champions that agreed to a follow-up visit and completed the follow-up survey. Among champions who made commitment 1, thirteen were assessed in a follow-up visit and nine were lost to follow-up. Among champions who made commitment 2, twenty-three were assessed in a follow-up visit and fourteen were lost to follow-up.

Attempts were made to engage all sites which committed to being champions in a follow-up visit and post-survey. The timing between completion of the initial and the follow-up visit was 26 days on average; sites engaged later in the project lifespan had a shorter time between the initial and follow-up visits. No association was found between the initial to follow-up visit time interval and whether champions completed their commitments.

At the initial visit, 37 (74.0%) champions committed to displaying COVID-19 and flu vaccine flyers in their store. Among the 23 champions who made this commitment and completed the post survey, only seven (18.9%) had them hanging in their site at follow-up. Compliance in hanging posters and flyers among businesses who refused the follow-up visit could not be assessed.

During initial visits, 17 (34.0%) champions committed to host a health department staff member at their site for a tabling event. Engagement staff followed up on those champions and scheduled tabling at the sites. However, after multiple scheduling attempts, four of the champions ultimately declined following through on their tabling requests. Tabling events were completed for the remaining 13 champions who committed to this activity during initial visits; during these events staff engaged directly with a total of approximately 245 residents. Two adjacent tabling events were scheduled simultaneously; during this time a COVID-19 mobile vaccine van was stationed outside of a champion business so that NYC Health Department staff could answer questions and refer interested residents directly to the mobile vaccination site.

At the initial visit, 22 (44.0%) champions committed to having vaccine conversations with their customers or community members. During the second visit, only 8 of these champions reported following through on this commitment (9 champions were lost to follow-up and 5 reported that they had not had conversations with their clients about the vaccines). As detailed in Table 2, most leaders focused on COVID-19 vaccines in their conversations; only two champions reported also having conversations about flu vaccines.

**Table 2:** Vaccine conversations: changing perceptions from initial visit to follow-up visit.

	Initial Visit N(%)	Follow-up Visit N(%)
**Vaccine hesitancy**^a^		
*What are the most common reasons that you have heard from your customers or other people in your community why they do not want to get the COVID-19 vaccine?*	30(100)	13(100)
Long-term effects are not known	3(10.0)	0(0.0)
Vaccines developed too quickly/waiting on full FDA approval	2(6.7)	2(15.4)
Might not do a good job of preventing transmission	4(13.3)	0(0.0)
Side effects	3(10.0)	1(7.7)
Not necessary if already had COVID	5(16.7)	2(15.3)
Current vaccines do not protect against new variants	1(3.3)	1(7.69)
Some other reason^c^	11(36.7)	4(30.8)
Don't know	10(33.3)	N/A
Did not talk to people since last meeting	N/A	5(38.5)
*What is the most common reason that you have heard from your customers or other people in your community why they do not want to get the Influenza vaccine?*		
Long-term effects are not known	2(6.7)	0(0.0)
Might not do a good job of preventing transmission	1(3.3)	0(0.0)
Side effects	3(10.0)	0(0.0)
Not necessary if already had flu	1(3.3)	0(0.0)
Some other reason^d^	23(76.7)	2(15.4)
Don't know	0(0.0)	N/A
Did not talk to people since last meeting	N/A	11(84.6)
**Confidence levels**^b^		
*When people share their concerns with you about COVID-19 or flu vaccines, how confident do you feel in talking with them about those concerns using information that you trust?*	30(100)	27(100)
Very Confident	13(43.3)	9(33.3)
Somewhat Confident	9(30.0)	4(14.8)
Neutral/No Opinion	7(23.3)	11(40.7)
Not Very Confident	1(3.3)	2(7.4)
Not Confident at All	0(0.0)	1(3.7)

a Responses from the initial visit represent businesses that were already having vaccine conversations with their clients. Results from the follow-up visit include only the champions that committed to having vaccine conversations and completed the post survey.

b Responses from the follow-up visit includes all champions that completed the follow-up survey including those that did not commit to having vaccine conversations.

c Responses for some other reason for COVID-19 vaccine hesitancy included public distrust in government, religion, misinformation on social media, or stubbornness during the initial visit. For the follow-up visit, other reasons for COVID-19 vaccine hesitancy included lack of trust of vaccine ingredients, lack of interest in the booster shot, and disapproval of mandates from government and businesses.

d Responses for some other reason for flu vaccine hesitancy was religion during the initial visit. As for the follow up visit, the two businesses that had flu vaccine conversations did not provide another reason for flu vaccine hesitancy but expressed that their clients had either already taken the flu vaccine or did not mention concerns about it.

### 3.2 Changing perceptions over time

#### 3.2.1 Vaccine hesitancy

As respected leaders in their community, site leaders were able to share perceptions about vaccine hesitancy. During the initial visit, among the 30 site leaders who reported that they had been having conversations with people about vaccines prior to the intervention, most did not perceive hesitancy in their community to be due to previously reported common concerns such as unknown long-term effects of the vaccine or the speed at which vaccines were developed [17]. Rather, a third of these leaders stated that they did not know the most common reasons for vaccine hesitancy, and 36.7% cited some other reason such as public distrust in government, religion, misinformation on social media, or stubbornness. During the follow-up visit, champions cited other reasons for COVID-19 vaccine hesitancy including lack of trust of vaccine ingredients, lack of interest in the booster shot, and disapproval of mandates from government and businesses.

#### 3.2.2 Confidence level of businesses

Twenty-two sites felt confident talking to people about COVID-19 and flu vaccine concerns before participating in the project. Notably, the overall confidence level among surveyed leaders decreased by the follow-up visit, which may be a result of conflicting messaging from government.

When asked during the follow-up survey if there was anything that would have helped them to feel more comfortable talking to their customers about the COVID-19 and flu vaccines, 100% of the respondents said “No”. Reasons cited included already feeling comfortable talking to people, the perception that COVID-19 is now over, and people are no longer willing to talk about it, and the sentiment that it is customers’ personal choice whether they should take the vaccine or not.

### 3.3 Community-level changes

Although the program sought to engage businesses/FBOs across four zip codes, engagement by zip code varied. Most champions were in 10466 and 10469; most tabling events were also completed there. As shown in Table 3, these two zip codes experienced higher percent increases in residents with at least one dose of a COVID-19 vaccine than the Bronx and NYC averages during the project period. The zip codes with less engagement in the program, 10470 and 10475, experienced percent changes in vaccinated residents closer to borough and citywide averages. Changes in flu vaccination rates during the project period were not available and therefore could not be assessed.

**Table 3:** Percent change in COVID-19 vaccination rate compared to champions’ commitments and program completion by zip code.

	Population^b^	Champions Commitments N(%)			COVID-19 Vaccination^a^		
		Number of Champions Committed	Number of Tabling Events Completed	Number of Champions who Finished Program^c^	Percent of all residents vaccinated pre-program (January 1, 2022)	Percent of all residents vaccinated post-program (May 1, 2022)	Percent Change in COVID-19 Vaccination Rate (January 1 to May 1, 2022)
10466	75,000	21(46.6)	6(46.2)	11(40.7)	68.3%	74.2%	+8.7%
10469	73,000	12(26.6)	4(30.8)	9(33.3)	69.0%	74.2%	+7.6%
10470	16,000	9(20.0)	2(15.4)	5(18.5)	75.6%	80.8%	+6.9%
10475	44,000	3(6.6)	1(7.69)	2(7.4)	73.1%	77.2%	+5.6%
Bronx	1,425,000	(--)	(--)	(--)	76.7%	82.7%	+7.2%
NYC	8,468,000	(--)	(--)	(--)	81.8%	87.0%	+5.6%

a Percent of residents all ages who have received at least one dose of a COVID-19 vaccine, per New York City Department of Health Citywide Immunization Registry [7].

b Population estimates rounded to the nearest thousand, per U.S. Census Bureau July 1, 2021 [2].

c Engaged in follow-up visit and completed follow up survey.

### 3.4 Champions’ experiences

When business leaders were asked about their overall experience as champions of health during the follow-up visit, they shared challenges as well as aspects of the program that worked well. Challenges included mixed messaging about COVID-19 and difficulty or discomfort in having conversations about the vaccines, detailed in Table 4. Champions also identified aspects of the program that were successful, noting that: the materials and resources were helpful, they were grateful to have access to PPE, and that they would be willing to talk to their customers about other health topics in the future. Out of the 27 businesses that completed the post survey, 16 were interested in future partnerships with the health department (4 declined and 7 hesitated).

**Table 4:** Results from the follow-up visit when champions were asked of their overall experience^a^ participating in the program.

**Mixed messaging about COVID-19 has led to misinformation and public distrust** • COVID-19 mandates and restrictions limited businesses • It is hard to change client views • Most people are not concerned about COVID-19 • The COVID-19 virus is overrated • COVID-19 is over
**Difficulty and discomfort in having vaccine conversations** • It is difficult to talk about COVID-19 with clients • Did not engage in COVID-19 and flu vaccine conversations • Not comfortable having COVID-19 conversations, unless started by clients
**Helpfulness of resources** • Flyers were very helpful in sharing information when clients needed them • Handed out flyers to clients when asked about COVID-19 • Distributed PPE to employees and clients
**Interest in promoting other health messaging** • Open to discussing other topics and resources in the near future • Do not mind the Health Department tabling in front of their business on other health topics • Interested in health promotion related to chronic illnesses (ex: health screenings, mental health resources) • Not interested in partnering with the health department in the future because they are busy

a Even though the detailing intended to discuss both COVID-19 and flu vaccines, engaged business leaders were more interested in talking about COVID-19.

## 4 Discussion

To mitigate vaccine hesitancy and increase vaccination rates, the Bureau of Bronx Neighborhood Health implemented a hyperlocal community detailing program to educate community members through influential business owners and faith-based leaders. The intervention successfully engaged many potential partners and developed a network of champions of health in the neighborhood. Greater percent increase in COVID-19 vaccination rates were observed in the zip codes with more involvement in the detailing program and these increases were higher than the Bronx and city averages.

### 4.1 Limitations

The evaluation of this effort is subject to several notable limitations. First, the evaluation was designed to show contribution of the program's efforts on overall vaccination rates, not attribution. Many simultaneous efforts, including the NYC Health Department's Public Health Detailing to providers and tireless efforts of community-based organizations in these neighborhoods, also seek to increase COVID-19 and flu vaccinations in the same zip codes. Citywide factors may also confound the relationship between this program and changing vaccination rates over time, particularly if those efforts were received and/or enforced differently by different communities. They include citywide public health messaging campaigns and the enforcement of vaccine mandates by private and public employers, which caused many residents to receive vaccinations to retain employment. Second, during the program duration, available information about the COVID-19 vaccinations continued to develop. The community detailing program focused on businesses that mostly reach adult clients, but some of these adults may be parents who received information about vaccines for youth. For this reason, the evaluation purposely considers vaccination rates for all-ages, but information about vaccine efficacy and safety for different age groups may have been received differently. At the time of this project, COVID-19 vaccines were not available for children under 4 years old. Finally, the small sample size of engaged champions limits ability to demonstrate effectiveness of the program.

### 4.2 Lessons learned

This project represented an intentional geographic departure from the Bureau of Bronx Neighborhood Health Action Center's typical catchment area in the South Bronx to concentrate resources in the Northeast Bronx, where COVID-19 and flu vaccine rates lagged behind other NYC neighborhoods. The businesses/FBOs engaged were new partners and allowed an opportunity to develop novel relationships. Since these business types often work with health inspectors, some had a preconceived perception that staff were visiting for enforcement purposes. In general, vaccine-related engagement in historically excluded communities requires repeat encounters and allocating ample time for discussion. In some cases, business owners actively refused participation in the survey, but the detailing teams were able to engage in long conversations with staff about their concerns and questions. Even when explicit objectives are not met (i.e., completed surveys, commitments as champions), important information can be shared, and misinformation or fear addressed. Despite hesitancy talking about vaccines, most sites were eager to receive PPE from staff; some even visited the bureau's building after the initial engagement to pick up additional PPE. Such benefits of this program extend beyond reported evaluation findings.

FBOs were not ideal for this detailing model. After multiple visit attempts, only 3 out of 32 originally identified FBOs engaged because many were closed or were conducting all worship virtually and none finished the program. Consequently, future iterations of this model should focus on businesses like barbershops, beauty salons, and other influential business types or attempt to engage FBOs when they return to in person service.

Due to the demographics of the Northeast Bronx community, having bilingual project staff was essential for engaging businesses, organizations, and community members. Many barbershops who became champions of health were only able to participate because the team could speak with the owner or manager in Spanish. Nevertheless, since many other languages are spoken in the community, detailing teams encountered language barriers when interacting with some sites which hindered the engagement level. Future detailing programs should leverage interpretation resources.

Although the intention of the project was to promote both COVID-19 and flu vaccinations, following the lead of business or FBO leaders, most engagement and discussion centered around COVID-19 vaccinations; therefore, future iterations might be best when focused on a single topic at a time, especially when dealing with controversial and politicized topics as they might dominate other topics during conversations. The program itself was limited by inconsistent COVID-19 public messaging by federal, state, and local government that made sustained engagement challenging. The lack of follow-through on champions’ commitments and interest in completing the follow-up visit and survey may be, in part, a result of the external political and social environment with respect to the COVID-19 pandemic. Many champions expressed that “COVID is over” or that people are tired of talking about COVID-19. Between the initial and follow-up visits, policies such as Key to NYC which required proof of COVID-19 vaccination to enter certain establishments and other local COVID-19 preventions such as mask mandates were suspended. Compounded with federal, state, and local messaging about “return to normalcy” and the end of the “pandemic phase”, this hindered efforts to engage with businesses and FBOs about vaccines. During this time, the existing vaccines were also demonstrated to be less effective in preventing infection against Omicron compared to prior variants. Sixteen businesses that initially committed to become champions refused the follow-up survey, potentially due to the shift in perceived importance of increasing vaccination rates. Nonetheless, zip codes with more engaged champions demonstrated increased COVID-19 vaccination coverage, indicating that business leaders can be influential champions for health in their communities.

### 4.3 Conclusion

The COVID-19 and flu community detailing project was the first of its kind in NYC. The project offered insight about ways to engage Bronx community members and partners in health messaging. Future planned iterations of the project include replicating the model with a chronic disease health lens and expanding potential champions to include other influential community leaders beyond business owners and FBOs.

## Statements of ethical approval

This work was determined not human subjects research by the NYC Health Department's Institutional Review Board and therefore not subject to ethical approval.

## Declaration of competing interest

The authors declare that they have no known competing financial interests or personal relationships that could have appeared to influence the work reported in this paper.

## Funding

This research did not receive any specific grant from funding agencies in the public, commercial, or not-for-profit sectors. The research was supported by funding from the New York City Tax Levy.

## Footnotes

1 Hair salons offer only hair services and products.

2 Beauty salons may offer hair services and products but also provide extended services including services related to skin health, facial aesthetics, foot care, nail manicures, aromatherapy, meditation, oxygen therapy, or mud baths.
